# Genomic characterization of the T-cell receptor loci in *Ambystoma mexicanum*

**DOI:** 10.3389/fimmu.2025.1656386

**Published:** 2025-09-30

**Authors:** Diana L. Pacheco-Olvera, Stephanie Saint Remy-Hernández, E. Ernestina Godoy-Lozano, Juan Téllez-Sosa, Humberto Valdovinos-Torres, Everado Curiel-Quesada, Constantino López-Macías, Jesus Martínez-Barnetche

**Affiliations:** ^1^ Departamento de Bioquímica, Posgrado en Ciencias Quimicobiológicas, Escuela Nacional de Ciencias Biológicas, Instituto Politécnico Nacional, Mexico City, Mexico; ^2^ Unidad de Investigación Médica en Inmunoquímica, Unidad Medica de Alta Especialidad (UMAE) Hospital de Especialidades, Centro Médico Nacional Siglo XXI, Instituto Mexicano del Seguro Social, Mexico City, Mexico; ^3^ Departamento de Parasitología, Posgrado en Ciencias Quimicobiológicas, Escuela Nacional de Ciencias Biológicas, Instituto Politécnico Nacional, Mexico City, Mexico; ^4^ Centro de Investigación Sobre Enfermedades Infecciosas, Instituto Nacional de Salud Pública, Cuernavaca, Morelos, Mexico

**Keywords:** Ambystoma, T-cell receptor (TR), TRA/TRD locus, TRB locus, pseudogenes, amphibians

## Abstract

**Background:**

Amphibians are valuable models for comparative immunology. In the caudate *Ambystoma mexicanum*, the architecture of immunoglobulin loci resembles that of the anuran *Xenopus tropicalis*, although some antibody gene features are absent. Evidence supports the presence of T lymphocytes in axolotl, the expression of T cell receptor alpha, beta, and delta chains, and a restricted diversity in the delta chain. Here, we describe the T cell receptor loci in the *A. mexicanum* genome and compare them with *X. tropicalis* and other tetrapods.

**Methods:**

T cell receptor loci were mapped and annotated in the *A. mexicanum* genome (UKY_AMEXF1_1) using reference sequences from axolotl, *X. tropicalis*, human, and mouse. Gene models were refined with RNA sequencing data from spleen, lung, and liver.

**Results:**

The T cell receptor alpha and delta locus in axolotl shows an overall conserved structure compared with other tetrapods. The alpha locus contained a higher number of variable genes than the beta and delta loci, with a predominance of functional genes (ratio 3.06). No gene encoding the pre-T cell receptor chain alpha was identified. The delta locus harbored two conventional variable genes, but no expression was detected in RNA sequencing data, suggesting pseudogenization. Neither delta chain diversity genes nor gamma chain elements were found in the genome or spleen transcriptome. The beta locus displayed structural similarity to that of other tetrapods and included five translocons with diversity, joining, and constant segments. One constant gene consisted of two exons encoding two constant domains. Functional variable genes predominated in the beta locus (ratio 3.6).

**Conclusion:**

Our study reveals conserved but distinctive features of axolotl T cell receptor loci, including restricted delta-chain diversity, absence of gamma chain and pre-T cell receptor alpha, and structural novelty in the beta locus. These findings provide new insights into the evolution of T cell receptors in amphibians and offer a genomic framework to explore the links between adaptive immunity and tissue regeneration in *A. mexicanum*.

## 1 Introduction

T-cell receptors (TR) recognize peptide antigens and other pathogen-derived molecules presented by antigen-presenting cells (APCs), a process essential for initiating adaptive cellular immunity. These receptors are expressed on the surface of T lymphocytes and enable specific antigen recognition through their variable extracellular domains. TR-mediated recognition is coupled to signaling via the CD3 complex (conformed by CD3γ, CD3δ, CD3ϵ chains), which transmits activation signals that drive T-cell activation, effector and memory differentiation, and clonal expansion (1, 2).

The general organization of TR genes has remained remarkably conserved throughout 400 million years of gnathostome evolution. Unlike the high variability of immunoglobulin loci, TR loci exhibit structural stability across vertebrates (3). A conventional TR is a disulfide-linked heterodimer composed of α and β chains, or γ and δ chains. Each of these four types of TR chains comprises two immunoglobulin superfamily domains: a membrane-proximal constant domain (C) and the antigen-binding variable domain (V). The V domains of TRβ and TRδ are assembled via somatic recombination of variable, diversity (D), and joining (J) genes, whereas the V domains of TRα and TRγ are assembled only by V and J genes (4). This recombination is mediated by recombination signal sequences (RSS) flanking each gene, consisting of a conserved heptamer and nonamer motif separated by either a 12- or 23-base pair spacer. According to the 12/23 rule, recombination typically occurs between one RSS with a 12-bp spacer and another with a 23-bp spacer, ensuring proper assembly of V(D)J junctions (5).

The TR α, β, γ, and δ chains are found across all jawed vertebrates, exhibiting significant conservation in both sequence and genomic arrangement. A distinctive feature is that the T cell receptor alpha (TRA) locus is embedded within the T cell receptor delta (TRD) locus (TRA-TRD locus), an organization conserved in all jawed vertebrates studied, including fish, amphibians, reptiles, birds, and mammals (6–11). However, the availability of non-model vertebrate genome sequences provides valuable insights into the distant origins of rearranging gene systems and their links to both adaptive and innate recognition processes (12). This approach has led to the identification of additional TR chains, such as the New Antigen Receptor (NAR-TCR) in sharks (13–15) and the T cell receptor μ (TRμ) chain in marsupials and monotremes, which originated from TRδ gene duplication during early mammalian evolution (16). Furthermore, in *Xenopus tropicalis*, the TRD locus contains canonical variable δ (Vδ) genes and VH-like genes termed VHδ, which are VH domains related to the variable domain of the immunoglobulin heavy chain, adapted as V-domains for TRδ chains (6). VHδ is also found in fish, birds, and monotremes. Overall, this highlights the remarkable evolutionary plasticity of TR evolution, likely due to selective pressure imposed by pathogen recognition (14, 17, 18).

Amphibians are well-suited models for comparative immune system analysis (1, 19), due to their key evolutionary relations as the first tetrapods, bridging aquatic vertebrates (e.g., fishes) and terrestrial vertebrates (20, 21). Their immune system comprises all major components of adaptive immunity, including T and B lymphocytes, immunoglobulins, and Major histocompatibility complex (MHC) molecules, enabling direct comparisons across both ancestral and derived vertebrate lineages. In addition, exhibit unique immunological features, such as unconventional TR gene arrangements or limited receptor diversity (6), offering insights into the evolutionary plasticity of the immune system. The study of TR loci in amphibians like *Ambystoma mexicanum* is particularly relevant because the immune system is increasingly recognized as a critical player in tissue repair and regeneration. Characterizing the genomic architecture and diversity of these loci not only informs our understanding of adaptive immunity in urodele amphibians but also provides a framework for investigating how immune components modulate regenerative processes. Such knowledge could facilitate the development of species-specific immunological tools, enhancing both biomedical research and conservation strategies.

*A. mexicanum*, a neotenic urodele amphibian endemic to the Mexico City valley, is an endangered species (22) and has one of the largest genome (32 Gb) among vertebrates sequenced to date (23). Previously, we characterized the immunoglobulin heavy (IGH) and lambda (IGL) loci in the *Ambystoma mexicanum*, finding that it shares the same general syntenic architecture with *X. tropicalis*, but lacks the kappa locus (IGK) and other antibody features described in *X. tropicalis* (24). Pre-genomic studies in *A. mexicanum* revealed the presence of T lymphocytes found in the spleen and thymus, as well as the presence of T cells expressing α, β, and δ chains (25–27). Of note, the junctional diversity of the TRδ chain is minimal (27, 28) and so far, no description of TRγ chains has been provided. Two genome assemblies are currently available for *A. mexicanum*: The AmbMex60DD genome assembly, based on a highly inbred laboratory strain *(d/d)*, a two-year-old leucistic male (23, 29) has 27,157 unmapped scaffolds and revealed several positional and orientation inconsistencies in the IGH locus, likely reflecting assembly errors (24). Recently, a new assembly, UKY_AMEXF1_1, generated from an F1 hybrid between *A. mexicanum* and *A. tigrinum*, both of wild origin has been publicly released. This assembly, which is currently the reference genome in GenBank, presents an improved chromosomal organization with 21 chromosomes and only 220 unmapped scaffolds (BioProject: PRJNA1165261), suggesting a more accurate annotation of complex loci.

To further investigate the germ-line structure of T cell receptors in *A. mexicanum*, we present here a genomic characterization and annotation of TR loci in the axolotl compared with *X. tropicalis*. This is one of the few amphibian species whose adaptive immune system has been extensively characterized at the genomic level (6, 30). In our previous analysis of the IGH and IGL loci in *A. mexicanum*, we reported the absence of certain components, such as the IGK locus and the pseudogenization of the IgF isotype (24). This feature had also been described in *X. tropicalis*. Building on these findings, one of the main objectives of the present study is to determine whether, as observed in the case of immunoglobulins, TR loci in *A. mexicanum* also exhibit missing or divergent components compared with other tetrapods.

## 2 Results

### 2.1 *Ambystoma mexicanum* TRA-TRD *locus*

**Figure 1:**
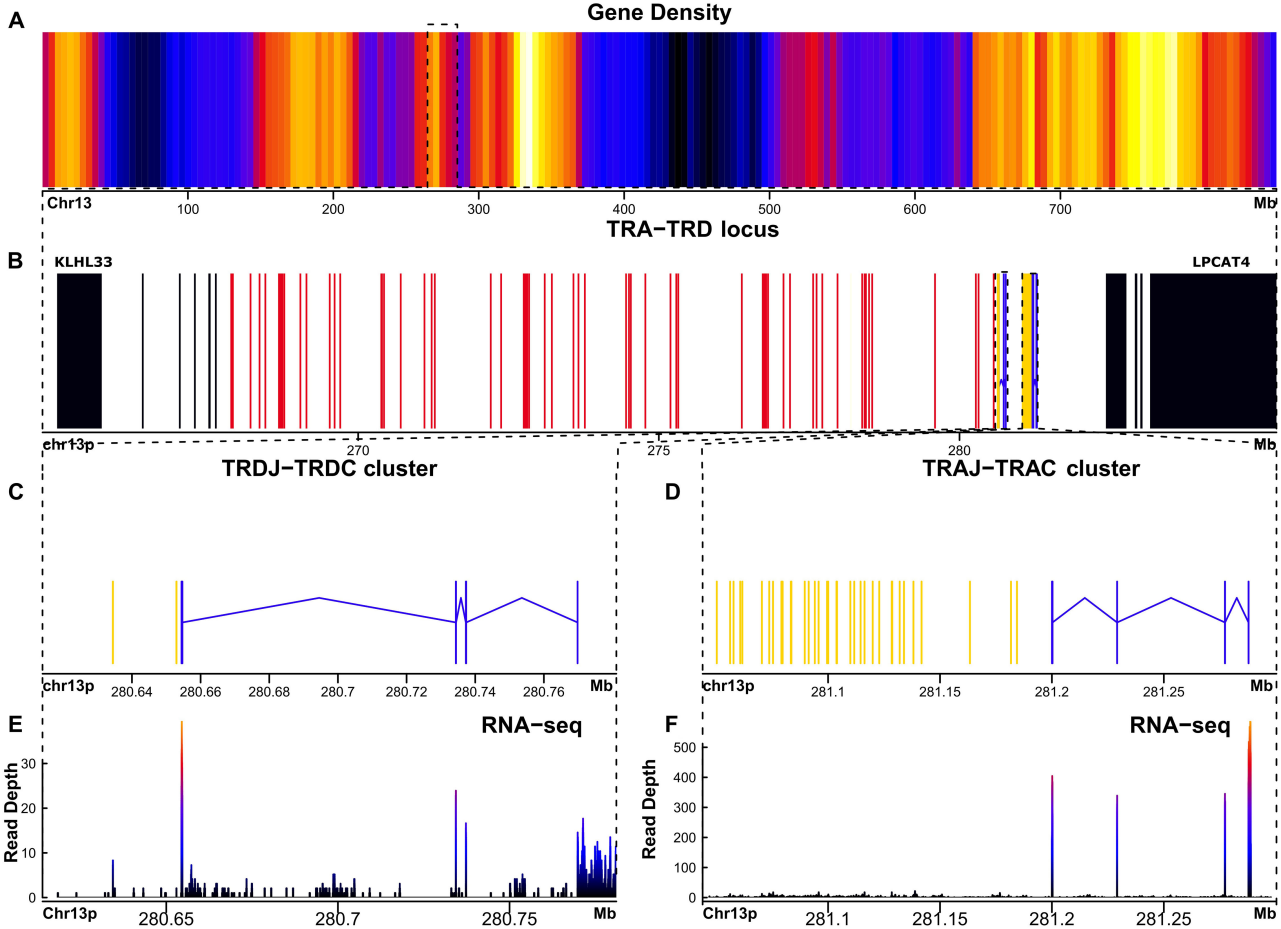
The TRA-TRD locus in *Ambystoma mexicanum* is located in the centromeric portion of chr13p (20.7Mbp). **(A)** Gene density plot of chromosome 13p, where the TRA-TRD cluster is highligted (box); dark blue colors indicate low gene density. **(B)** Overview of the whole TRA-TRD locus (264.6-285.3 Mbp), showing non-TR genes (black) in proximal flank, TRAC and TRDC genes (blue), TRAJ and TRDJ genes (yellow), and TRAV and TRDV genes (red). **(C)** Close-up of the TRDC-J gene cluster (267.8-280.7 Mbp). **(D)** Detailed view of theTRAJ cluster (281-281.2 Mbp). **(E)** Spleen RNA-seq coverage histogram of the TRDC-J region. **(F)** Spleen RNA-seq coverage histogram of the TRAC-J region. Note that in the E and F panels the color intensity shifts from blue to red whith read counts increasing in the spleen transcriptome.

A phylogenetically conserved feature of the TRA and TRD locus organization in vertebrates is that both loci are closely embedded (6, 14, 31, 32) near the centromere of chr13. Accordingly, in *A. mexicanum*, the TRA and TRD locus are closely linked, with some TRD genes nested within the TRA locus. The TRA-TRD locus in *A. mexicanum* is located in chromosome 13p: 264.6-285.3 Mbp (size 20.7 Mbp) (**Figures 1A, B**) (**Supplementary File 1**; **Supplementary Figure S1**; **Supplementary File 2**; **Table 1** and Glossary) and is not interrupted by gaps. In *X. tropicalis*, the TRA-TRD locus was mapped to chromosome 1 (0.72-1.24 Mbp; size 0.52 Mbp) (6). We identified 61 T cell receptor alpha variable genes (TRAV), 46 of which are functional, flanked by canonic Recombination Signal Sequences of Variable genes (V-RSS) with 23-bp spacers, corresponding to Functional/Pseudogenes (F/P) ratio of 3.06. We identified 36 T cell receptor alpha join genes (TRAJ), of which 33 are functional. All TRAJ genes encode the canonical FGXG motif and have a 12-bp spacer and conserved heptamer and nonamer in their Recombination Signal Sequences of Join genes (J-RSS) (**Supplementary File 1**; **Supplementary Figure S2**). We found three TRAJ pseudogenes, one of which (TRAJ_036) is intercalated within the intron of the T cell receptor alpha constant gene (TRAC). Its J-RSS lacks the conserved 5´-CAC-3´ motif in the RSS heptamer, which is required for Recombination activator gene (RAG) recognition during V(D)J recombination (33). The pseudogenes TRAJ_015 and TRAJ_023 contain a frameshift (**Supplementary File 2**; **Table 1**, Glossary and GFF file). A single TRAC gene with canonical exons, including cytoplasmic (M2), transmembrane (M1), and C-Ig domain exons (**Figures 1D-F**; **Table 1**), has three glycosylation sites in 42-45 (NDTE), 72-75 (NDTQ), and 106-109 (NESF). In the transmembrane region (TM), residues Cys225, Glu237, Arg251, Lys256, Asn261, Tyr265, and Trp269, which interact with CD3, are mostly conserved in *A. mexicanum* except for Arg251, which is replaced with Lys (34, 35). The connecting peptide motif (FETDXXLN), another important site in the TM region for the transduction of activation upon antigen recognition (36), is well conserved in the axolotl. Furthermore, Cα sequence alignment across human, mice, opossums, frogs, and axolotls reveal limited conservation of the AB loop in *X. tropicalis* and *A. mexicanum* regarding mammals (**Supplementary File 1**; **Supplementary Figure S3**).

**Table 1:** Summary of total number and percentage of functional and pseudogenes of V, D, J and C genes found in the TRA-TRD locus mapped in Chr 13.

Gene type	Number of functional genes	Percentage of functional genes (%)	Number of pseudogenes	Percentage of pseudogenes (%)	Total
TRAV	46	75.4	15	24.59	61
TRAJ	33	91.6	3	8.3	36
TRAC	1	100	0	0	1
TRDV	1	50	1	50	2
TRDD	There is no evidence of the presence of TRDD genes				
TRDJ	2	100	0	0	2
TRDC	1	100	0	0	1

The TRD locus harbors two conventional T cell receptor delta variable genes (TRDV) flanked by a canonical V-RSS with a 23-bp spacer; however, one of them is not transcribed based on spleen, lung, and liver RNA-seq data, suggesting it is a pseudogene. Only two T cell receptor delta join genes (TRDJ) were found (**Figures 1C-E**) (**Supplementary File 1**; **Supplementary Figure S1**). Both TRDJ genes appear to be functional; however, the consensus J motif (FGXG) encoding the di-glycine bulge is not present in Jδ1 (FKKG), whereas Jδ2 retains the canonical sequence. The J-RSS is conserved in both genes, with a 12-bp spacer (**Supplementary File 1**; **Supplementary Figure S4**). No Dδ genes or their corresponding recombination signal sequences of diversity (D-RSS) were identified. The exon organization of the single T cell receptor delta constant gene (TRDC) was found with canonical exons, including cytoplasmic (M2), transmembrane (M1), and C-Ig domain exons (**Figure 1C**; **Table 1**). This TRDC exon encodes an N-glycosylation site (NSSS, pos 36-39).

**Figure 2:**
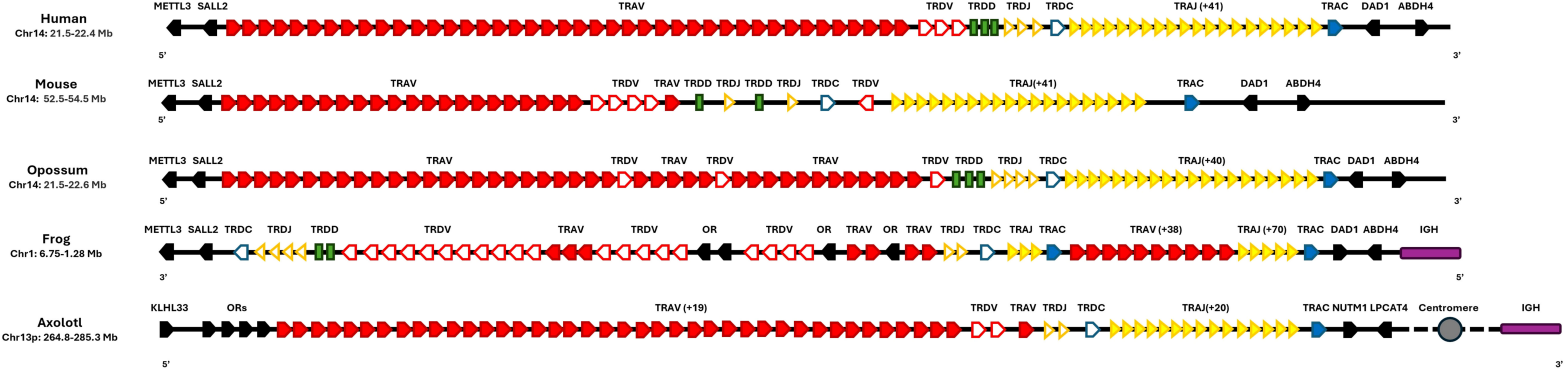
Synteny of the TRA-TRD locus in *Ambystoma mexicanum* compared to other tetrapods. Schematic representation of TRA-TRD locus in human (*Homo sapiens*, GRCh38.p14), mouse (*Mus musculus*, GRCm39), opossum (*Monodelphis domestica*, ASM229v1), frog (*Xenopus tropicalis*, UCB_Xtro_10.0), and axolotl (*A. mexicanum*, UKYF1_1) are shown. Solid-filled symbols represent the TRA locus, while open symbols correspond to the TRD locus. Constant regions are indicated in blue, J gene cluster is depicted in dark yellow, and V clusters are highlighted in red. D genes are displayed as green rectangles, and non-TR genes are depicted in black. Interestingly, in the axolotl, the IGHC locus *(*purple) is not linked to the TRAD cluster as observed in *X. tropicalis* and spans across the centromere (depicted as a gray circle). The figures are not to scale, and the same scheme is applied to all species for consistency. Gene orientation in mammals and axolotl is 5’-3’and in *X. tropicalis* is 3’-5’.

In all studied vertebrates so far, the TRA and the TRD locus are genetically linked (5, 33–35) in different vertebrates such as frog, human, mouse, and opossum. In all of these species, the TRA-TRD locus is flanked by the METTL3, SALL2, DAD1, and ABHD4 genes (5, 7, 8, 34, 36–38). However, the *A. mexicanum* locus is flanked by NUMP and LPCAT4 in the centromere direction and the KLHL33 gene in the telomeric direction (**Figure 2**; **Supplementary File 3**: **Table 2**).

**Table 2:** Summary of total number and percentage of functional and pseudogenesof V, D, J and C genes found in the TRB locus mapped in the Chr 3p.

Gene type	Number of functional genes	Percentage of functional genes (%)	Number of pseudogenes	Percentage of pseudogenes (%)	Total
TRBV	18	78.26	5	21.7	23
TRBD	5	100	0	0	5
TRBJ	17	100		0	17
TRBC	6	100	0	0	6

### 2.2 *Ambystoma mexicanum* TRB *locus*

**Figure 3:**
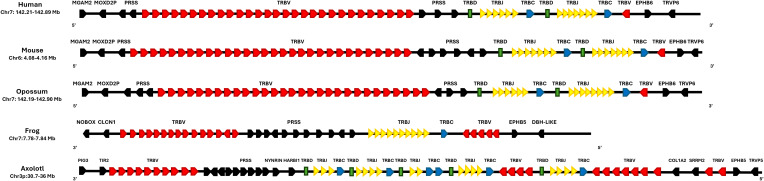
Synteny of the TRB locus in *Ambystoma mexicanum* compared to other tetrapods. Schematic representation of the TRB locus in human (*Homo sapiens*, GRCh38.p14), mouse (*Mus musculus*, GRCm39), opossum (*Monodelphis domestica*, ASM229v1), frog (*Xenopus tropicalis*, UCB_Xtro_10.0), and axolotl (*A. mexicanum*, UKYF1_1). The TRBC genes (constant regions) are shown in blue, the TRBJ cluster in yellow, and the TRBV cluster in red. TRBD genes are depicted in green, while non-TR genes are represented in black. The figure is not to scale, and the color scheme is consistent across all species for clarity. Gene orientation in mammals is 5’-3’and in amphibians is 3’-5’.

**Figure 4:**
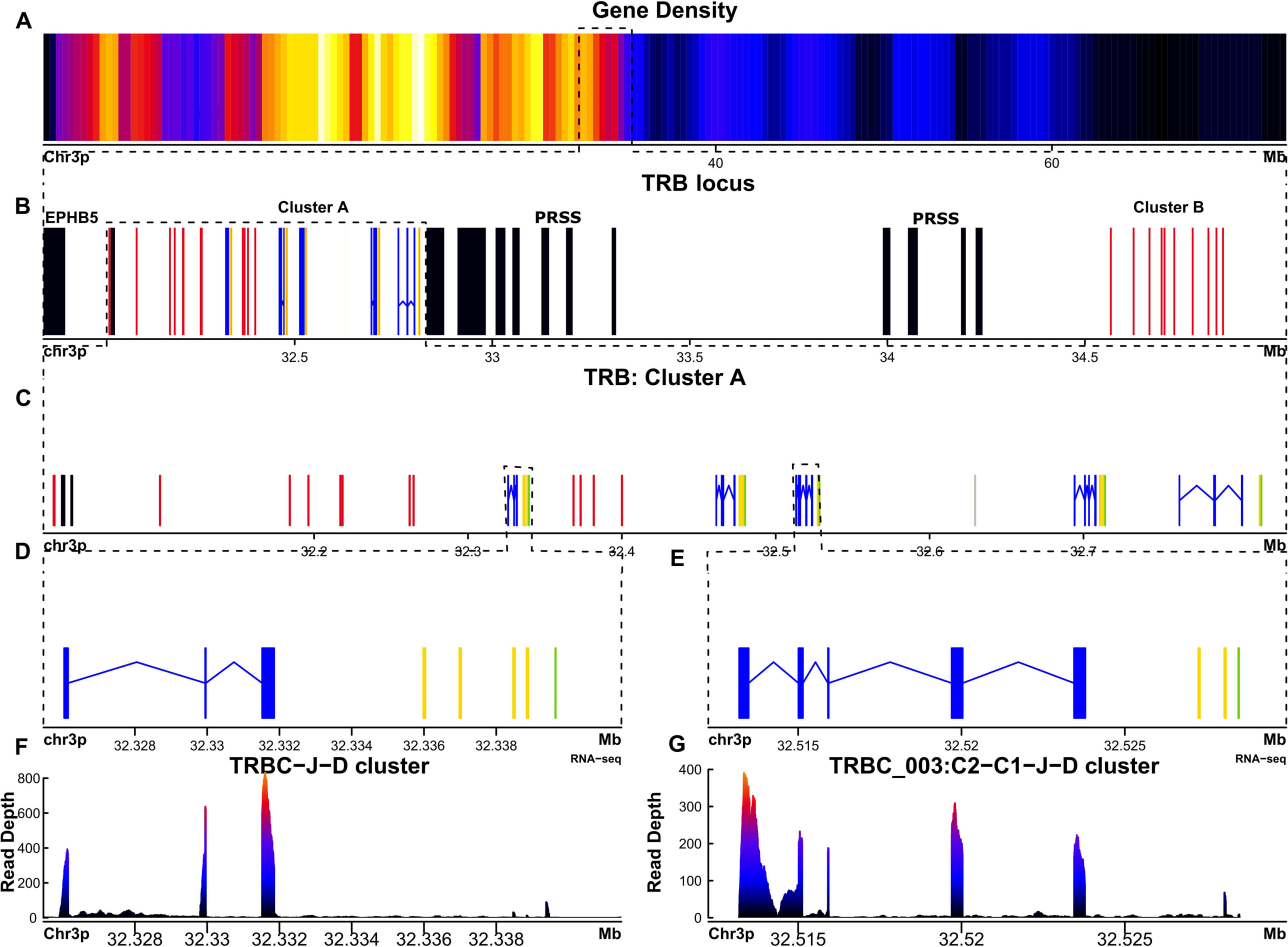
The TRB locus of *Ambystoma mexicanum* is located in the telomeric portion of chromosome 3p (4.82 Mbp). **(A)** Gene density plot of chromosome 3, where the TRB locus is encoded (highlighted with a box). Dark blue regions indicate areas of low gene density. **(B)** Overview of the TRB locus (30.03-34.85 Mbp), showing non-TR genes in black. Proximal and distal flanking genes include TRPV and PRSS, respectively.Gaps are represented in gray. TRBC genes are shown in blue, TRBJ genes in yellow, TRBD genes in orange, and TRBV genes in red. **(C)** Zoomed view (32.03–32.9 Mbp) of the TRB cluster A, showing detailed gene organization. **(D)** Close-up of the TRBC-J-D functional genes. **(E)** Close-up view of the TRBC_003 gene, which includes two Cβ-domain exons. **(F)** Spleen RNA-seq coverage histogram of the TRBC-J-D region, showing transcriptional activity. **(G)** Spleen RNA-seq coverage histogram of the TRBC-C2-J-D region, indicating transcription levels in this area. Note that in the **E** and **F** panels the color intensity shifts from blue to red whith read counts increasing in the spleen transcriptome.

The T cell receptor beta (TRB) locus in *X. tropicalis* has not been previously described. We mapped the *X. tropicalis* TRB locus to chr7p (3–8 Mbp), flanked by the DPH-like and EPHRIN genes towards the centromere, and NOBOX and CNCL in the telomeric direction (**Figure 3**). A trypsin gene cluster (PRSS) was found between T cell receptor beta join genes (TRBJ) and T cell receptor beta variables clusters (TRBV) (**Figures 4A-C**) (**Supplementary File 1**; **Supplementary Figure S5**; **Supplementary File 3**: **Table 2**). A similar organization is observed in the human and mouse TRB locus; however, in *X. tropicalis*, the locus is inverted regarding the EPHRIN gene.

In *A. mexicanum*, the TRB locus was identified on chromosome 3p (30.03-34.85 Mbp, size 4.82) (**Figures 4A, B**; **Supplementary File 2**; **Table 1** and GFF file) and contains a single gap in position 32.62 Mbp. The trypsin (PRSS) gene cluster divides the TRB locus into a canonical locus towards the centromere (referred to hereafter as cluster A), and a TRBV gene cluster (cluster B) towards the telomere (**Figure 4B**). Cluster A compromises five tandem TRBC-TRBJ-TRBD translocons and a TRBV gene cluster comprising 13 TRBV genes (**Figure 4C**). Cluster B contains 10 TRBV genes. Overall, there are 18 functional TRBV genes (9 in cluster A and 9 in cluster B) to F/P ratio of 3.6. Additionally, there are 5 functional T cell receptor beta diversity genes (TRBD). All TRBD genes exhibit 12-pb and 23-pb spacers. Recombination signal sequences of diversity genes (D-RSS) at their respective flanks (**Supplementary File 1**; **Supplementary Figure S6**). Furthermore, we found 17 functional TRBJ genes encoding the FGXG motif, with canonical 12-bp spacer J-RSS (**Supplementary File 1**; **Supplementary Figure S7**).

We identified five functional T cell receptor beta constant genes (TRBC), one associated with each translocon (**Figures 4D, E**). Spleen, lung, and liver RNA-seq data revealed that all functional genes are actively transcribed (**Figures 4F, G**). The functional genes feature the conserved Trp at position 41, the Leu at position 86, and the two characteristic Cys residues of the constant genes at positions 23 and 104 (Based on IMGT numbering of human TRBC1). Genes TRBC_001, 002, 004 and 005 share the typical TRB gene structure composed by a single Cβ exon with a glycosylation site (TRBC_001 100-103 (NITV), TRBC_002 100-103 (NITV), TRBC_004 7-10 (NVTQ), TRBC_005 51-54 (NRTK)), a M1 and M2 exons encoding a linker peptide and the transmembrane region, respectively (37–39). The TRBC_003 is unusual because it comprises two Cβ-domain exons, (TRBC_003_1 and TRBC_003_2) (**Figures 4E-G**; **Table 2**). The TRBC_003_1 exon encodes for a glycosylation site (4–7 NITQ), whereas the TRBC_003_2 exon lacks predicted N-glycosylation sites.

In mammal Cβ-domain, the FG loop is implicated in the interaction with the CD3 complex (40). Sequence alignment of the Cβ domains from human, mouse, opossum, frog, and axolotl revealed the absence of the FG loop in all *X. tropicalis* and *A. mexicanum* Cβ. In all compared amphibian Cβ domains, the proline residue at position 232 is conserved, except in TRBC_003_2, where it is replaced by an alanine (Ala 228) (**Supplementary File 1**; **Supplementary Figure S8**) (40, 41).

### 2.3 No evidence of an *Ambystoma mexicanum* TRG *locus*

**Figure 5:**
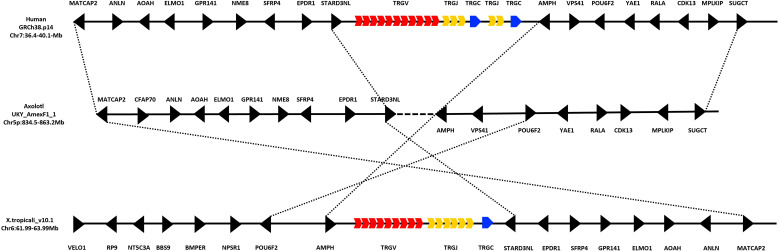
Absence of the TRG locus in the *Ambystoma mexicanum* genome (UKYF1_1). Schematic representation illustrating the absence of the TRG locus. In humans, the TRG locus is located on chromosome 7 (36.4–50.1 Mb), while in *Xenopus tropicalis*, it is located on chromosome 6 (61.99–63.99 Mb). The V cluster is shown in red, the J cluster in yellow, and the constant (C) region in blue, regardless of functionality. Non-TR genes are depicted in black. In *A. mexicanum*, analysis of chromosome 5p (834.5–863.2 Mb) reveals a complete absence of the TRG locus. However, strong synteny is observed with the genomic neighborhood found in both humans and *X. tropicalis*. The proximal flanking genes include *AMPH*, *POU6F2*, *NPSR1*, *BMPER*, *BBS9*, *NT5C3A*, *RP9*, and *VELO1*, while the distal flanking genes are *STARD3NL*, *EPDR1*, *SFP4*, *GPR141*, *ELMO1*, *AOAH*, *ANLN*, and *MATCAP2*. Notably, the orientation of these flanking genes is inverted in *X. tropicalis* compared to *A. mexicanum* and humans, which share not only the same gene content but also a conserved gene orientation. This suggests that, despite the loss of the TRG locus in *A. mexicanum*, the surrounding genomic architecture remains highly conserved.

We use BLAST and HMMER alignment-based search tools, either in the genome or in spleen RNA-seq transcriptome data, we found no evidence of the existence of the T cell receptor gamma locus (TRG) in *A. mexicanum*. In *X. tropicalis*, the TRG locus is located on chromosome 6 (Chr6: 63.4-63.6 Mb), flanked proximally by the *STARD3NL, EPDR1, SFP4, GPR141, ELMO1, AOAH, ANLN*, and *MATCAP2* genes and the *AMPH, POU6F2, NPSR1, BMPER, BBS9, NT5C3A, RP9*, and *VELO1* genes at the distal flank. In humans, the TRG locus is located on chromosome 7 (Chr7: 38.24–38.36 Mb), with a genomic neighborhood like that of *X. tropicalis*, although the orientation of the flanking genes is inverted. In this case, the proximal flanking genes are *AMPH, POU6F2, NPSR1, BMPER, BBS9, NT5C3A, RP9*, and *VELO1*, while the distal flanking genes are *STARD3NL, EPDR1, SFP4, GPR141, ELMO1, AOAH, ANLN*, and *MATCAP2*. In contrast, *A. mexicanum* exhibits a genomic architecture similar to that observed in humans, although located on chromosome 5p (Chr5p: 834.5–863.3 Mb), with no evidence of the TRG locus in this region (**Figure 5**; **Supplementary File 3**; **Table 2**).

### 2.4 V-intron length

V genes are composed of two exons: Exon 1 encodes the L1 part of the leader peptide, whereas exon 2 encodes the L2-part of the leader peptide and the V-region. Both exons are separated by the V-intron that typically ranges in size from 80 to 250 bp (42). In axolotl, the average V-intron length was 758 bp for TRA, 142 bp for TRD (considering two genes), and 237 bp for TRB (**Supplementary File 2**; **Table 1**, columns L-O). We analyzed the distribution of V-intron lengths in functional TRAV (P=0.0002) and TRBV (P=0.51) genes. No significant differences were observed between functional and non-functional TRBV genes. In contrast, a significant difference was detected in TRAV, suggesting that most TRAV genes with long V-introns are functional (**Supplementary File 1**; **Supplementary Figure S9**).

To investigate the relationship between V-intron length and V gene functionality in the TRA and TRB loci of *A. mexicanum*, we assessed whether the presence of long introns (>650 bp) was more frequent in non-functional TRAV and TRBV genes, as previously reported for the IGH and IGL loci (24). Fisher’s exact test was applied to the TRAV and TRBV genes. For TRAV, the analysis revealed that the odds of finding a long intron in a non-functional gene were 0, resulting in an odds ratio of 0.0 (p=0.01035; 95% CI: 0.0–0.66). This suggests a significant depletion of long introns among non-functional TRAV genes. In the case of TRBV, no significant association was found (p=1; 95% CI: 0.0–233.15), likely due to the limited number of observations, making statistical comparison uninformative.

### 2.5 *Ambystoma mexicanum* PTCRA locus

**Figure 6:**
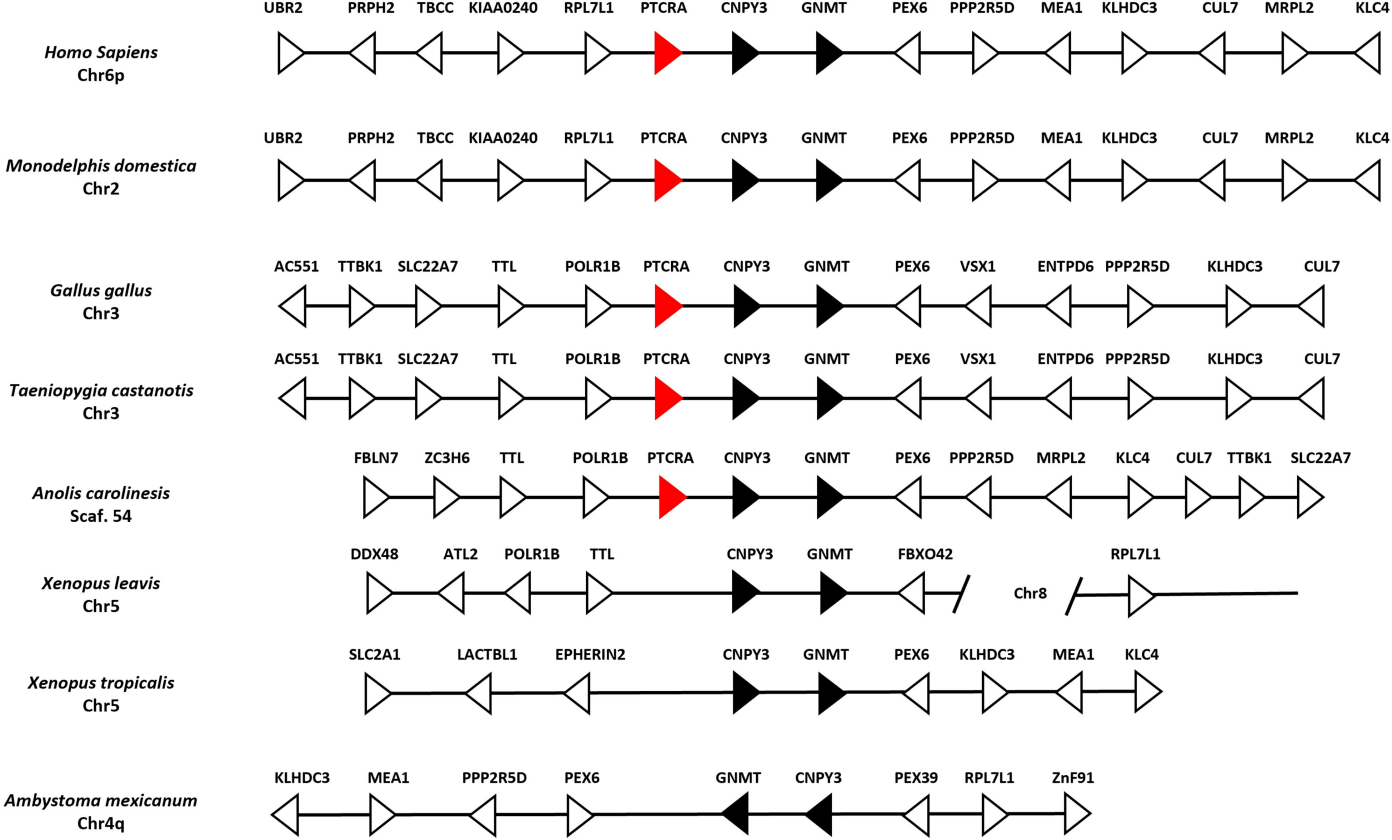
Absence of the PTCRA locus in the *Ambystoma mexicanum* genome (UKYF1_1). Schematic representation of the PTCRA locus across representative genomes. Black arrows indicate syntenic genes, white arrows represent non-syntenic genes, and red arrows depict the PTCRA gene ortholog. In mammals (*Homo sapiens* and *Monodelphis domestica)*, there is perfect synteny in the locus. In sauropsids, such as *Gallus gallus*, *Taeniopygia castanotis* (chicken and zebra finch) loci are syntenic; in *Anolis carolinensis* (lizard), only *POLR1B, TLL* upstream and downstream *CNPY3*, *GNMT, PEX6*, and *CUL7* are conserved. In *X*. *tropicalis* synteny is observed but there is no evidence of PTCRA. In the case of *A. mexicanum*, we identified all neighboring genes of the PTCRA locus; however, a rearrangement of the entire chromosome is observed, leading to the absence of PTCRA.

In humans and mice, the pre-TCRα participates in αβ T cell early development in association with the TRβ chain at the surface of thymocytes. It is known that this receptor is absent in non-mammalian species such as *Xenopus* spp. and *Danio rerio (*Zebrafish) *(* 43). We performed BLAST and HMMER searches in the *A. mexicanum* genome and transcriptome and found no evidence of a PTCRA ortholog. Additionally, synteny analysis of the PTCRA locus across multiple species revealed conserved synteny between *X. laevis, X. tropicalis*, and *A. mexicanum*, confirming the absence of PTCRA in *A. mexicanum* in contrast to mammals and sauropsids (**Figure 6**).

## 3 Discussion

The UKY_AMEXF1_1 genome assembly of *A. mexicanum* enabled a comprehensive characterization of its TR loci, supported by an increased N50 = 1.5 compared with the AmbMex60DD genome version N50 = 1.2. The overall structure of the TRA-TRD locus is conserved, but the TRD locus exhibits strikingly low combinatorial diversity, and the TRG locus is absent, suggesting that *bona fide* TRγδ T cells may be lacking in axolotls. The TRB locus displays a conserved structure with tandem duplications of the TRBD-TRBJ-TRBC translocon, including a particular TRBC gene with two Cβ domains. As in *X. tropicalis*, the PTCRA gene is absent.

These findings provide important insights into the genomic organization and evolutionary constraints of TR loci in urodele amphibians. The limited diversity of TRD genes and absence of the TRG locus highlight unique features of the axolotl adaptive immune system, which may have implications for understanding T-cell function in regeneration and immune response. Overall, this work establishes a foundation for comparative immunogenomic studies across amphibians and other vertebrates.

Here we confirm such findings. We found similarities in the overall structure of the TRA-TRD locus, as well as a strikingly low combinatorial diversity of the TRD locus and the absence of the TRG locus, implying the absence of *bona fide* TRγδ T cells in axolotl. As for the TRB locus, we describe a conserved structure, with tandem duplications of the TRBD-TRBJ-TRBC translocon and a particular TRBC gene composed of two Cβ domains. As in *X. tropicalis*, the axolotl genome also lacks the PTCRA gene.

*A. mexicanum’s* exceptionally large genome (32 Gb) posed significant challenges for its assembly. The AmbMex60DD (white strain, *d/d*) version was released in 2021, based on 30× genomic coverage of 28 chromosomes and 27,157 unmapped scaffolds. This assembly presented several positional and orientation inconsistencies in the IGH locus, likely reflecting assembly errors (24). The current genome assembly (UKY_AMEXF1_1) was released in 2024 and has an increased genomic coverage (48×), 21 chromosomes, and only 220 unmapped scaffolds, suggesting that a more accurate complex loci annotation can be achieved. However, as with AmbMex60DD, no publicly available data on local coverage is currently provided for UKY_AMEXF1_1. Therefore, it is not possible to assess coverage-based metrics for individual TR genes.

In agreement with our previous analyses using the AmbMex60DD assembly, the overall expression patterns of TR genes did not substantially change in the present study (**Supplementary File 4**; **Supplementary Table 3**). Remarkably, we identified two genes, TRAV_060 and TRDV_001, that exhibit a structurally complete configuration, including the SP, canonical donor and acceptor splice sites, conserved methionine’s at positions 23 and 104, and canonical RSS. However, neither of these genes showed detectable expression in the analyzed tissues. Conversely, two additional genes, TRAV_025 and TRBV_012, also retained an intact genomic organization and displayed transcriptional evidence of the V gene, yet lacked detectable expression of the corresponding SP. According to our classification criteria, these cases were therefore categorized as pseudogenes, since evidence of both V gene and SP expression was required to consider a gene as functional.

Despite the mentioned limitations, the remarkably stable of the TRA-TRD locus across species, maintaining a consistent genomic architecture for over 340 million years of evolutionary history, highlights strong evolutionary constraints on its organization (6). The fact that TRA and TRD remain genetically linked in all examined species (14, 44–46) reinforces the functional importance of their physical association. This overall structure, conserved in *X. tropicalis*, alligators, birds, and mammals. In all of these species, the TRA-TRD locus is flanked by the *METTL3, SALL2, DAD1*, and *ABHD4* genes (6, 9, 14, 16, 45, 47, 48) suggesting that this syntenic arrangement may be critical for maintaining locus integrity. In contrast, the distinct configuration in *A. mexicanum*; located on chromosome 13p, near the centromere and flanked by KLHL33, likely reflects a lineage-specific chromosomal rearrangement that preserved the internal gene order, indicating that positional changes do not necessarily disrupt locus function (**Figure 2**) (**Supplementary File 1**; **Supplementary Figure S1**).

In *A. mexicanum*, the residue equivalent to mammalian TRα Arg251 is replaced by Lys, a substitution that is unlikely to affect its functional contact with CD3δ, given the chemical similarity of both residues (34, 35). The connecting peptide motif in the Cα transmembrane region is conserved, maintaining its role in signal transduction from the αβ heterodimer to the CD3/ζ complex (36).

Interestingly, as in *X. tropicalis*, *A. mexicanum* lacks the AB-loop, a structural feature essential for CD3ϵδ contact and T cell activation in mammals (34, 49). The absence of the AB-loop in amphibians may reflect a distinct co-evolutionary trajectory of TR and CD3 complexes compared to mammals (50).

We confirm the restricted diversity of germline genes previously reported for the TRD locus. As described by André, et al. (28) the cause of such restricted diversity is determined by a single functional TRDV and two TRDJ genes, but significantly, we confirm the absence of TRDD genes in the germline. As in other tetrapod TRD loci, the V and J genes are flanked by 23-bp and 12-bp spaced RSS, respectively. Hence, direct TRDV-TRDJ junctions do not violate the 12/23 rule (51). TRDV-TRDJ junctions have been described in a subset of human acute lymphoblastic leukemias (52). To our knowledge, *A. mexicanum* is the first vertebrate capable of non-pathological direct TRDV-TRDJ recombination.

A notable feature of the TRA-TRD locus in the axolotl is that the only TRDV gene is of the conventional Vδ type and not of the VHδ type, in striking contrast to *X. tropicalis*, which contains 14 VHδ and 2 conventional Vδ genes (6). The VHδ type is widely distributed among non-placental mammals and other vertebrates (9, 10, 15, 16, 48, 53, 54), and it remains to be determined if this type of element was lost in all or some caudates or if it was never present.

Compared with the TRα and β chains, the presence of TRγ and TRδ chains shows more heterogeneity across different taxa (32). In scaled reptiles, there is an absence of the TRD and TRG loci (55). Although the syntenic blocks flanking the TRG locus in *Xenopus* were identified in *A. mexicanum*, no genomic and transcriptomic evidence of the presence of the TRG locus was found. These results indicate that axolotl lacks true γδ T cells. It remains to be determined the functional role of the single TRδ chain, and if it pairs at all with itself or with another chain, but due to its invariant structural nature, it may function essentially as a Pattern Recognition Receptor (PRR), similarly to BTNL/Btnl family of innate γδ TR (56).

In this study, we updated and expanded the genomic annotation of the TRB locus located on chr3p previously described using cDNA libraries (57–59). The TRB locus in tetrapods is generally organized into TRBD-TRBJ-TRBC units, resembling the organization of the lambda chain locus (60). For instance, sheep possess three TRBD-TRBJ-TRBC tandem units, while rabbits, mice, and humans have two (42, 61, 62). In comparison, *X. tropicalis* has a simpler configuration with a single unit. Remarkably, *A. mexicanum* displays a more complex arrangement, consisting of five tandem TRBD-TRBJ-TRBC units.

In conventional TRβ chains, the Cβ domain interacts with CD3 through the FG-loop, contributing to signal transduction upon MHC-peptide recognition. In mammals, mutations in the FG-loop alter the CD8+ and CD4+ T cell proportions, and it is associated with a poor antigen response (63). Our study in axolotl is in agreement with a previous report by Kim, et al., reporting the absence of the FG-loop in non-mammal vertebrates (**Supplementary File 1**; **Supplementary Figure S8**) (40, 41). We identified a novel TRBC gene (TRBC_003) that features two Cβ domains, along with transmembrane and cytoplasmic domains, each encoded by a separate exon. The TRBC_003 gene is actively transcribed in the spleen and associated with putatively functional Dβ and Jβ genes. Moreover, in the distal membrane exon (TRBC_003_1), there is a conserved Pro232, which is a relevant position of the FG-loop in mammals with a single N-glycosylation site. In contrast, in the proximal membrane exon (TRBC_003_2), there is an Ala in the 228 position and no N-glycosylation sites. This structural evidence suggests that the proximal exon may have arisen from a duplication of the distal exon. Whether a TRβ product of the TRBC_003 gene pairs with a TRα chain, how it interacts with CD3, and how MHC-peptide interaction takes place remain as open questions.

Genome sequencing of F1 crosses between *A. mexicanum* and *A. tigrinum* have revealed genomic regions of high polymorphism (64, 65), however the TR loci are outside these regions. We consider that spurious contributions of allelic variation to gene count are likely minimal. Moreover, genome information derives from a single individual, and our findings may thus fail to capture the extent of intra-species variability, particularly copy number variation in Adaptive Immune Receptor Repertoire (AIRR) loci, which are well documented in mice, macaques, and humans (66–68), that may explain discrepancies between the UKY_AMEXF1_1 and the AmbMex60DD assemblies.

We observed that many TRAV genes in *A. mexicanum* possess notably long V-introns, some exceeding 1,000 bp; and are considerably larger than IGH and IGL V-introns in *A. mexicanum* (24). In the *Gallus gallus* genome, the average TRB V-intron length is 400 bp (69). The biological implication of longer TR V-introns is uncertain. Long introns have been reported to impose evolutionary costs by increasing the energetic demand due to the greater nucleotide investment and extended transcription time required. Additionally, they may compromise the fidelity of mature mRNA and expand the sequence space available for allelic variation and aberrant splicing (70). Intron length has also been suggested to play a functional role in evolutionary dynamics (71). Comeron and collaborators (72) proposed that extensively long introns may enhance the efficiency of natural selection by alleviating Hill-Robertson (HR) interference, a phenomenon where selection acting on linked loci reduces selective efficacy. In this context, long V-introns may act as spacer regions that decouple selective pressures acting on neighboring functional elements.

Moreover, HR interference might prevent their elimination via purifying selection if these introns are linked to active V genes. Consequently, such introns could facilitate the emergence of alternative splicing events or the production of non-functional transcripts, potentially affecting the expression and functionality of the antigen receptor repertoire (70, 73, 74). Collectively, these findings reinforce the idea that introns do not merely represent structural and energetic burdens but may also play key roles in gene generation, conservation, and diversification; particularly in immune-related loci such as those encoding T cell receptors.

It is noteworthy that the functional-to-pseudogene ratio in the *A. mexicanum* TRB is 3.6 and 3.06 in the TRA locus, compared with 0.9 and 2.7 in the IGH locus *(*AmbMex60DD) *(* 24) and UKY_AMEXF1_1^1^ assemblies, respectively. Within the framework of the “birth-and-death” model of T and B cell receptor gene evolution (75), higher ratios may indicate a slower accumulation of pseudogenes, potentially reflecting more recent functional gene birth events, stronger purifying selection, or lower rates of gene inactivation in T cell receptors compared with B cell receptors. While such differences could be stochastic, they may also reflect distinct selective pressures acting on these repertoires. The comparison between genome versions further shows that improvements in assembly quality can refine gene counts and alter calculated ratios, underscoring the importance of high-quality chromosome-level genome assemblies and thorough manual curation of AIRR loci for robust evolutionary inferences (76).

Its absence in amphibians implies that early αβ T cell development proceeds via alternative mechanisms, possibly involving different surrogate chains or signaling pathways. This reinforces the hypothesis that PTCRA originated as an amniote-specific innovation, rather than an ancestral gene lost independently in teleosts and amphibians. Identifying how amphibians compensate for the absence of PTCRA could provide insights into the evolution and diversification of T cell developmental programs in vertebrates.

The review and characterization of immune components in *A. mexicanum* are essential for understanding the cellular processes in which cells interact dynamically and persistently. These components have been shown to play a key role in modulating such interactions in other vertebrates with comparable regenerative capacity, influencing both the persistence of immune responses and the regulation of mechanisms underlying the regeneration of complex structures such as limbs, tail, heart, retina, and spinal cord. However, further studies are needed to clarify the specific role of T cells in this process in *A. mexicanum* (77–80).

In conclusion, the *A. mexicanum* TRB locus exhibits greater diversity than the TRA and TRD loci. Notably, no evidence of bona fide γδ T cells was found. This study leaves open questions regarding the composition of T-cell subpopulations and the pairing of TR chains, particularly the δ chain in the absence of the γ chain in axolotl. The presence of two constant domains in TRB_003 warrants further investigation to clarify their role in antigen recognition, functionality, and pairing with the α chain. Such insights are crucial to understanding the impact on this endangered species, which already presents a marked deficit of heterozygosity, reflecting substantial inbreeding and increasing vulnerability to infectious diseases (22).

This work provides valuable insights for comparative evolutionary analyses in tetrapods and advances our knowledge of immune response in caudate amphibians. Moreover, it may aid in solving specific questions regarding the role of acquired immunity in the regulation of the immune response implicated in tissue regeneration (81).

## 4 Methods

### 4.1 *Ambystoma mexicanum* genome and transcriptome data

The published sequence of the *A. mexicanum* haploid genome (UKY_AmexF1_1; GenBank assembly: GCF_040938575.1) was generated from an adult female F1 hybrid (isolate Amex_F1_6; BioSample SAMN41071122) derived from a cross between a female *A. mexicanum* (isolate Mex_15411; BioSample SAMN43142723) and a male *A. tigrinum* (isolate Tig_M23; BioSample SAMN43142724). The phased haploid assembly has 48x coverage was sequenced using PacBio and Illumina HiSeq and Hi-C data (82). The UKY_AmexF1_1 assembly consists of 21 chromosomes and 220 unplaced scaffolds, with a scaffold N50 of 1.5 Gb.

The corresponding genome annotation file GCF_040938575.1_UKY_AmexF1_1_genomic.gff.gz; and gap positions GCF_040938575.1_UKY_AmexF1_1_genomic_gaps.txt.gz, were retrieved from https://ftp.ncbi.nlm.nih.gov/genomes/all/GCF/040/938/575/.

To validate gene models, we used previously published RNA-seq and transcriptome data available in the NCBI SRA database. Specifically, RNA-seq coverage bigWig (BW) files from spleen, liver, and lung (SRR15610271, SRR15610267, SRR15610267), obtained from the NCBI FTP repository (https://ftp.ncbi.nlm.nih.gov/genomes/all/GCF/040/938/575/GCF_040938575.1_UKY_AmexF1_1/RNASeq_coverage_graphs/), were used as visual support for manual curation without further processing. Additionally, transcriptome datasets (BioProject PRJNA378970) from spleen, liver, and lung (SRR5341570, SRR5341572, SRR5341571) (23) were aligned to the genome using STAR (83) with default parameters and a maximum intron size of 3000 bp. Gene-level quantification was performed using the –quantMode GeneCounts option.

Mapping statistics for each dataset and assembly including input reads, uniquely mapped reads, spliced alignments, mismatch rate per base, and reads discarded for being too short. RNA-seq datasets were obtained from adult tissues (NCBI SRA: SRR5341570: spleen; SRR5341572: liver; SRR15610267: lung) and aligned to both AMEX_F1_1 and AmbMex60DD assemblies. These statistics provide a benchmark for expression analysis and support the reliability of TR gene annotation across assemblies (**Supplementary File 4**; **Supplementary Table 3**).

### 4.2 TR loci mapping

Reference sequences (cDNA) for TRA, TRD, TRB, and TRG loci from *X. tropicalis*, *X. laevis*, and *A. mexicanum* were obtained from NCBI (https://www.ncbi.nlm.nih.gov/). These sequences were used to map the TRA, TRD, and TRB loci using TBLASTX and Exonerate (EST2genome alignment model). Hits with significant alignment scores (e-value < 1.0E-05 for BLASTX, score > 100 for Exonerate) were exported as GFF3 files. These annotations were visualized and manually curated using the Integrative Genomics Viewer (IGV) (84). To complement homology-based annotation and minimize the risk of missing novel or lineage-specific V(D)J genes, we developed a custom pipeline to detect RSSs according to the canonical 12/23 rule. The workflow comprised: (i) BLAST alignments with bitscore filtering and conversion to GFF to identify scaffolds or chromosomes of interest; (ii) Exonerate-based exon and gene detection; (iii) RSS search using HMMER with tbl-to-GFF conversion; (iv) redundancy reduction across gene and exon annotations; (v) overlap analyses to confirm V genes and their signal peptides; (vi) refinement of V gene and RSS-J coordinates with Miniprot (protein-to-genome aligner); and (vii) identification of candidate D genes based on flanking 5′ and 3′ RSSs. This pipeline was applied to the TRA, TRD, and TRB loci in the AmbMex60DD assembly to uncover additional putative V, D, and J genes. The search database was built from TR gene models previously described in *X. tropicalis* and *X. laevis*. Genes identified through RSS were integrated with homology-based results and manually curated. To refine annotation, the AmbMex60DD-derived sequences were subsequently aligned to the latest reference genome (AMEX_F1_1), yielding the final TR loci annotation. All TR genes were named by a provisional numeric identifier. All our annotations are compliant with the IUIS T-cell Receptor and Immunoglobulin Nomenclature Sub-Committee, except for the fact that individual gene coverage and loci coverage are not publicly available (85).

### 4.3 Definition of V, D, and J functionality

Functionality assessments were performed based on IMGT (86) criteria. To classify a V, D, or J gene as functional (F), each coding region was required to have an open reading frame, proper splicing sites, and recombination signals with 12/23 spacers. For V-exons, the presence of conserved residues Cys23, Trp41, Trp52, and Cys104 was mandatory. Genes were classified as pseudogenes (P) if they contained stop codons, lacked leader peptide exon and/or RSS and were frame-shifted. For J-exons, the di-glycine bulge (FGXG) was required.

### 4.4 Search for TRDD and TRBD genes

We constructed a Hidden Markov Model (HMM) profile with the HMMER3 (-hmmbuild option) (87) to represent the sequence structure of the genes of TRBD and their associated RSS’s. This profile was generated with multiple sequence alignments based on TRBD genes from *A. mexicanum* previosuly published by Fellah (59). The genes were flanked at the 5’ by a 12 bp-spaced RSS, and at the 3’ end by a 23 bp-spaced RSS. The same HMM profile was subsequently used to search for the TRA-TRD locus.

### 4.5 PTCRA gene search

A multiple sequence alignment of PTCRA orthologs described by Smelthy et al. (43) was used to build an HMM profile with the HMMER3 (-hmmbuild option). This probabilistic model captures evolutionary changes in conserved amino acids across related sequences (87). The resulting HMM profile was applied to the *A. mexicanum* proteome using -hmmsearch. Additional searches were performed with the PFAM model PF15028 for PTCRA.

For synteny analysis, we first identified PTCRA-flanking genes in human, opossum, birds, reptiles, and frogs. Orthologous regions were then located in *A. mexicanum*, followed by manual curation of the surrounding genes to ensure the annotation was correct.

### 4.6 Intron length analysis

V-intron length was calculated from the exon coordinates of the respective locus annotation file (**Supplementary 2**; **Table 1**, P column). Due to the presence of abnormally long intron, we used a non-parametric Wilcoxon rank-sum text to compare intron lengths between functional and pseudogene V genes. Enrichment of long introns in functional genes was further evaluated using Fisher’s exact test in R.

## Data Availability

The original contributions presented in the study are included in the article/**Supplementary Material**. Further inquiries can be directed to the corresponding authors.

## Footnotes

1 Saint Remy-Hernández S, Pacheco-Olvera DL, Godoy-Lozano EE, Miguel-Ruiz JA, Téllez-Sosa J, Valdovinos-Torres H, et al. An update of immunoglobulin loci in Ambystoma mexicanum. (2025).
