# Supplementary material for: Genomic characterization of the T-cell receptor loci in Ambystoma mexicanum
Source: Front Immunol. 2025 Sep 30;16:1656386. doi: 10.3389/fimmu.2025.1656386 (PMC12529555; doi:10.3389/fimmu.2025.1656386)
Supplement: Supplementary file 1 [file DataSheet1.pdf]

Supplementary figures

Index

Figure S1: Representation of the TRA-TRD cluster in the *Ambystoma mexicanum* genome (UKY_AMEXF1_1) in Chr13p.

Figure S2: Structural analysis of the *Ambystoma mexicanum* TRAJ genes.

Figure S3: Multiple alignments of TRAC.

Figure S4: Structural analysis of the *Ambystoma mexicanum* TRDJ genes.

Figure S5: Representation of the TRB cluster in the *Ambystoma mexicanum* genome (UKY_AMEXF1_1) in chr3p.

Figure S6: Structural analysis of TRBD genes in chr3p.

Figure S7: Structural analysis of the *Ambystoma mexicanum* TRBJ genes.

Figure S8: Multiple alignments of TRBC.

Figure S9. TRA-TRB V-intron.

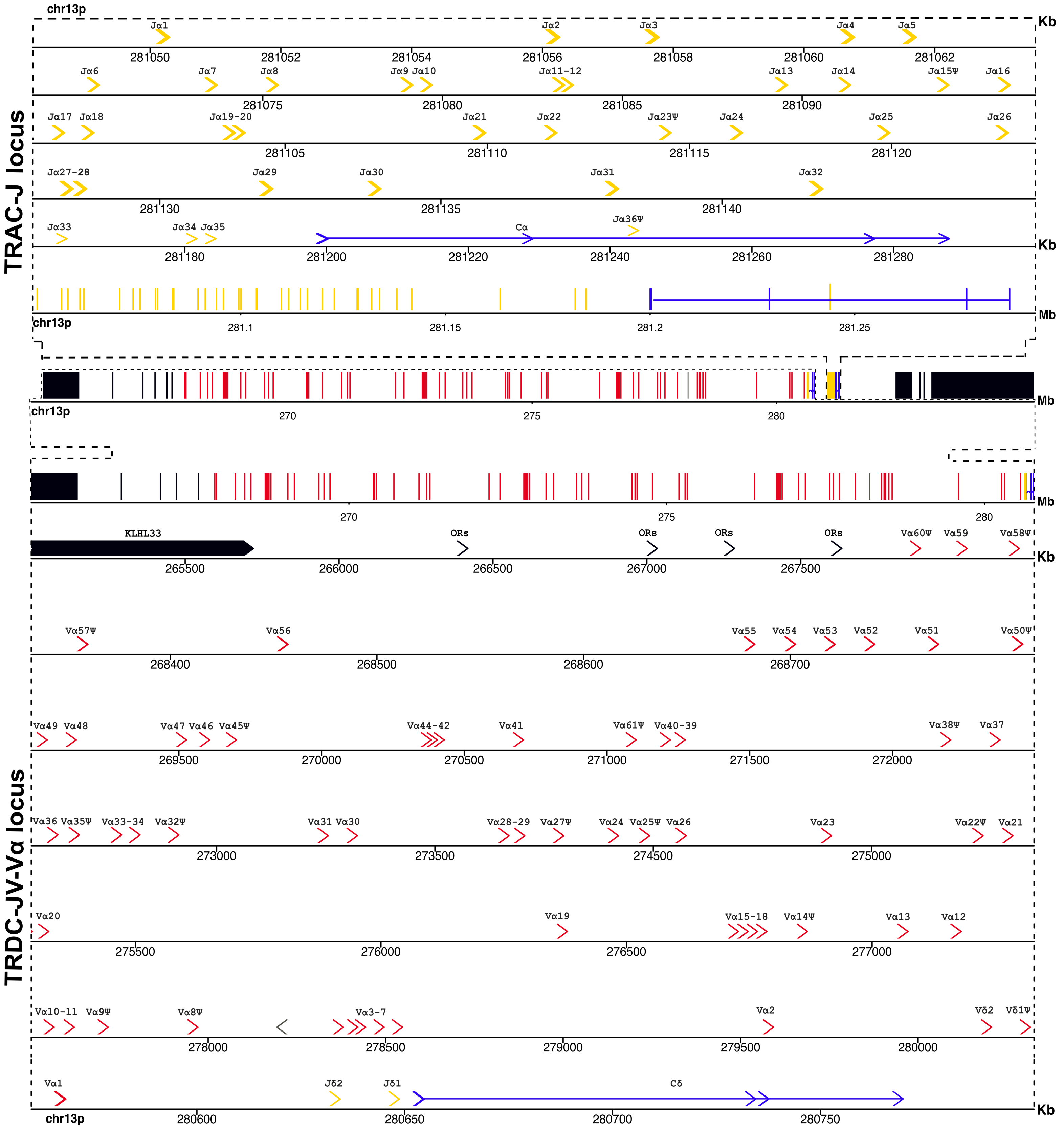

Figure S1. Representation of the TRA-TRD cluster in the *Ambystoma mexicanum* genome (UKI_AMEXF1_1) in chr13p. TRAD *locus* (chr13p: 264.6-285.3 Mbp). Genes in black are non-TR genes, gaps in gray, TRAC and TRDC in blue, TRAV and TRDV genes in red, and finally TRAJ and TRDJ genes in yellow. In the zoomed region of the TRDC-JV cluster 265-280.78 Mbp), the exons of the delta constant gene are shown, along with the TRAV cluster and the two TRDV genes. Pseudogenes are denoted by the letter Ψ. On the other hand, in the zoomed region of the TRACJ cluster (281.04-281.06 Mbp), the complete TRAJ cluster is displayed. The orientation of each gene is indicated by an arrow. Gene orientation is direct chain 3-5´.

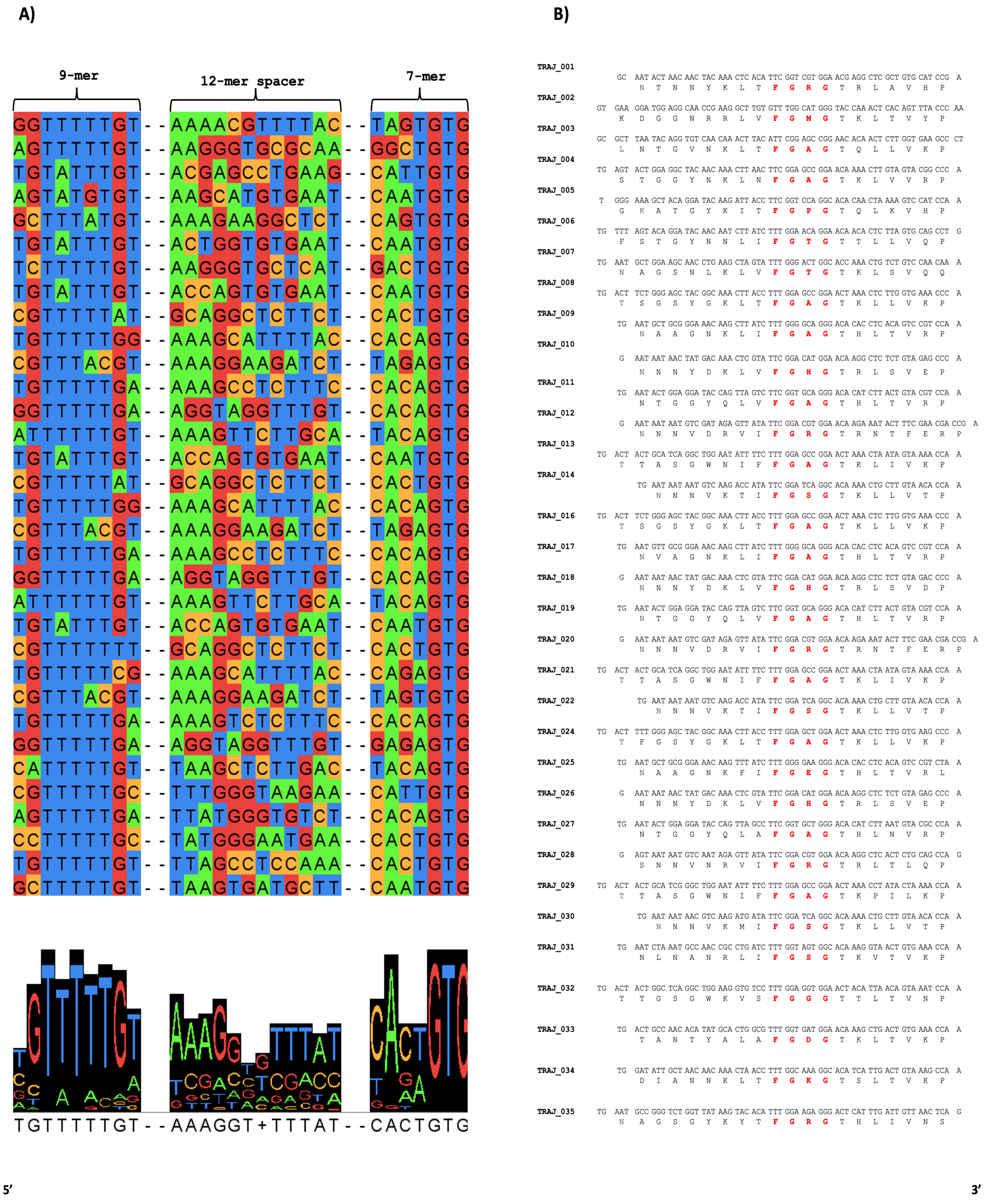

Figure S2. Structural analysis of the *Ambystoma mexicanum* TRAJ genes. A) Nucleotide alignment sequences of the RSS showing the conserved heptamer and nonamer separated by a 12 bp spacer, considering that the sequences shown correspond to the direct chain 5'- 3' **B)** Alignment nucleotide and amino acid sequences of the 33 identified TRAJ functional genes, the conserved FGXG motif, characteristic of the TRA J-REGION, is in red.

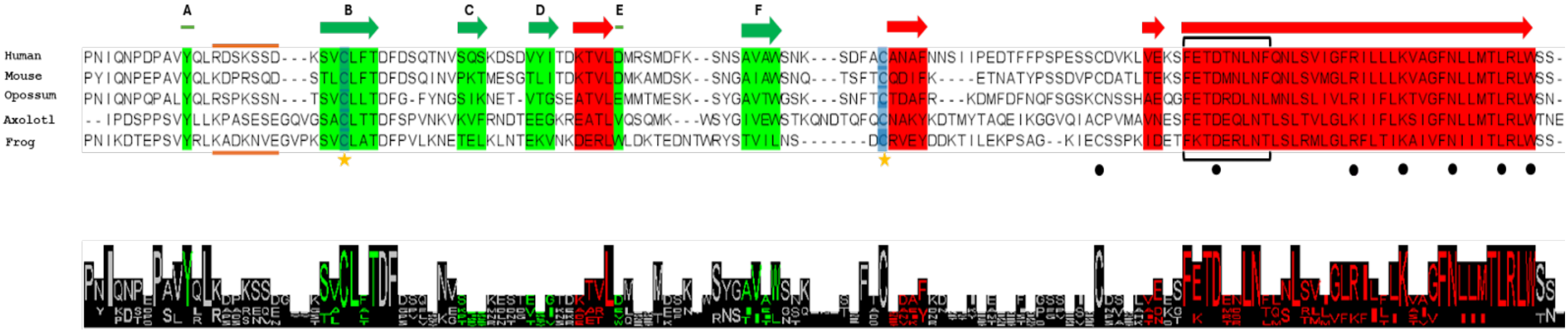

Figure S3. Multiple alignments of TRAC. Sequence comparison of the TRAC AB loop regions among various species (human, mouse, opossum, axolotl, and frog). The AB loop is indicated with orange lines. In mammals, this region is well-conserved (Arg129, Asp130, Ser131, Lys132, Ser133, Ser134, and Asp135). The yellow stars indicate the cysteines contributing to the disulfide bond. The bracketed region defines the connecting peptide motif (FETDXXLN), an important site in the transmembrane region for the antigen response. With black circles, the conserved amino acids throughout all species in the Cα transmembrane region are indicated. The arrows in green show the strands, and the red ones are the α-helices in the secondary conformation.

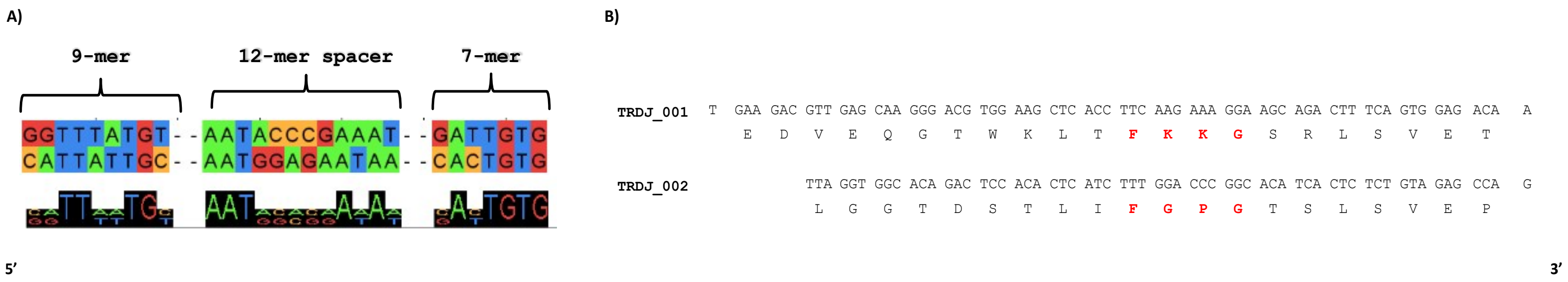

Figure S4. Structural analysis of the *Ambystoma mexicanum* TRDJ genes. A) Nucleotide alignment sequences of the RSS showing the conserved heptamer and nonamer separated by a 12 bp spacer, considering that the sequences shown correspond to the direct chain 5'-3'. **B)** Alignment of nucleotide and amino acid sequences of the two identified TRDJ functional genes; however, one of them did not have the canonical glycine bulge (FKKG) characteristic of the TRD J-REGION.

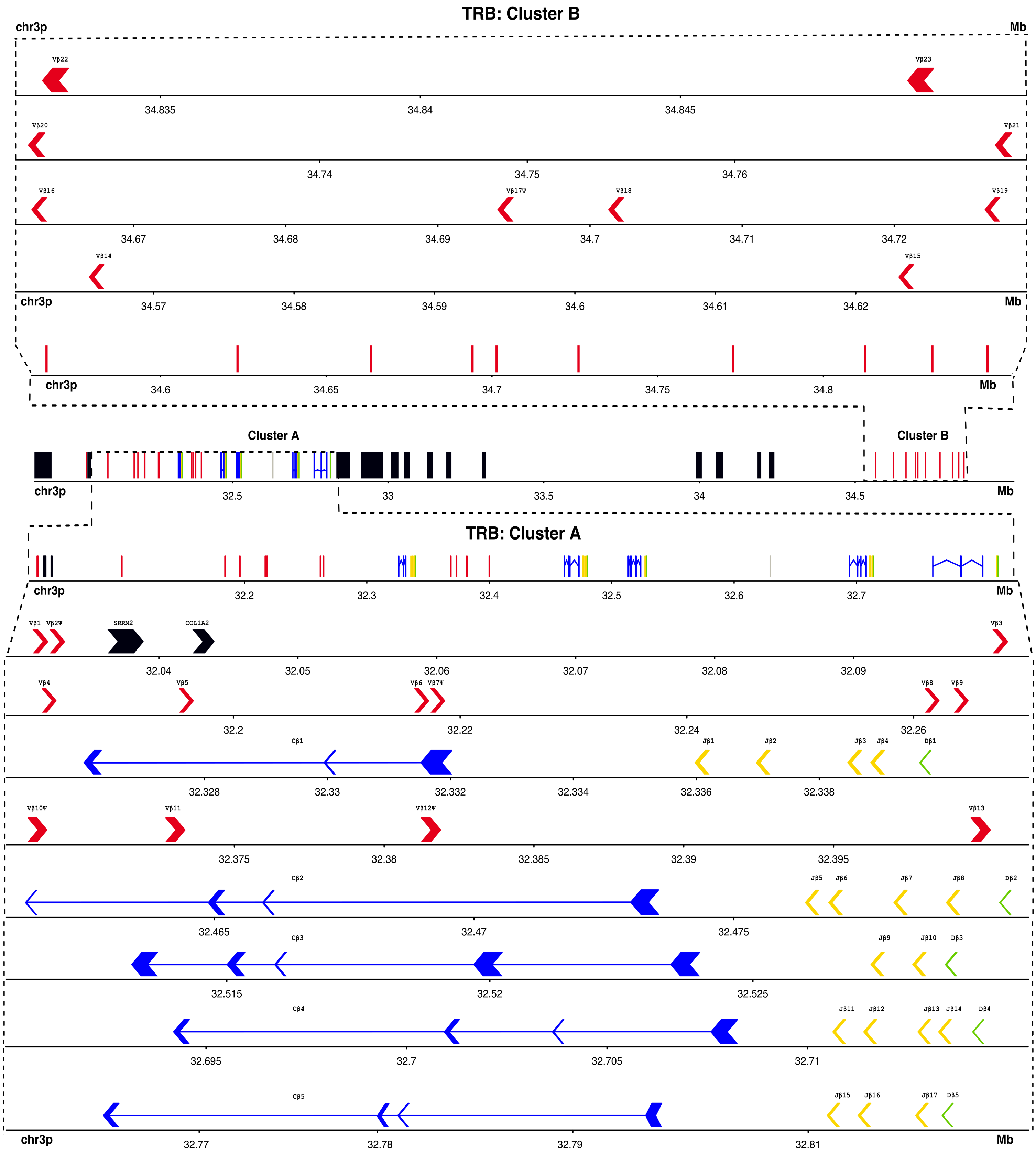

Figure S5. Representation of the TRB cluster in the *Ambystoma mexicanum* genome (UKY_AMEXF1_1) in chr3p. TRB *locus* (chr3p: 30.03-34.85 Mbp). Genes in black are non-TCR genes, TRBC in blue, TRBV genes in red, TRBJ genes in yellow, and TRBD genes in orange. In the zoomed region of cluster A 32.03–32.9), the VDJ and C genes are displayed. Pseudogenes are denoted by the letter Ψ. In the case of cluster B (34.56-34.85 Mbp), a unique cluster of downstream V genes is presented, interspersed among the trypsin genes within this *locus*. The arrow indicates the orientation of each gene. Gene orientation is direct chain 3´-5´.

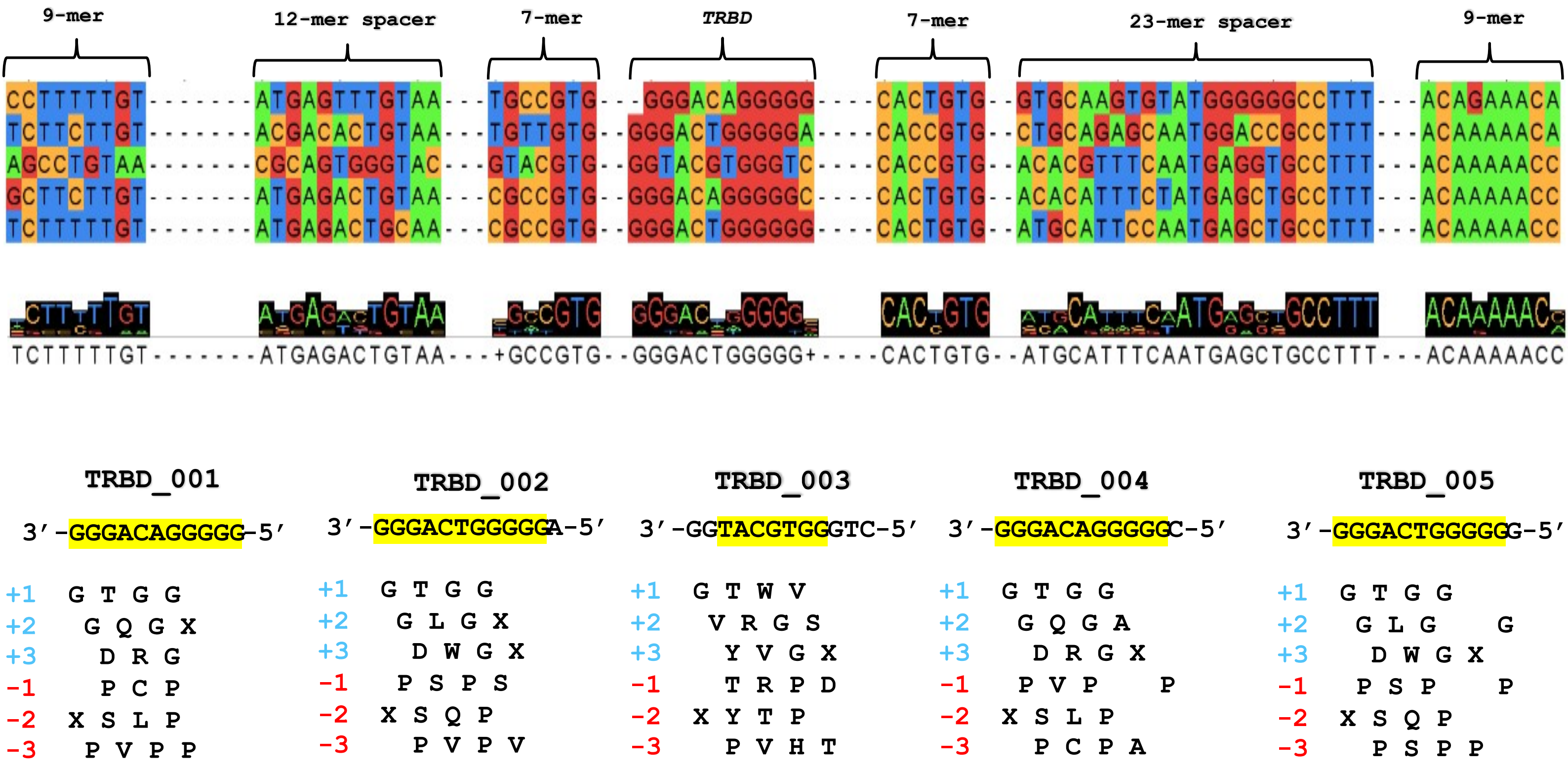

Figure S6. Structural analysis of TRBD genes in chr3p. Multiple alignments of the five identified TRBD plus the RSSs in both flanks show the conserved 9-mer and 7-mer separated by a 12-mer spacer on both flanks—sequence translation of TRBD genes. The nucleotide sequence is shown in lower case. Identical positions to TRBD sequences described by Fellah, *et al.,* 2001 are highlighted in yellow (TRBD_001 = Dβ1, TRBD_002 = Dβ2/Dβ4, TRBD_003 = Dβ3, TRBD_004 = Dβ1, and TRBD_005 = Dβ2/Dβ4).

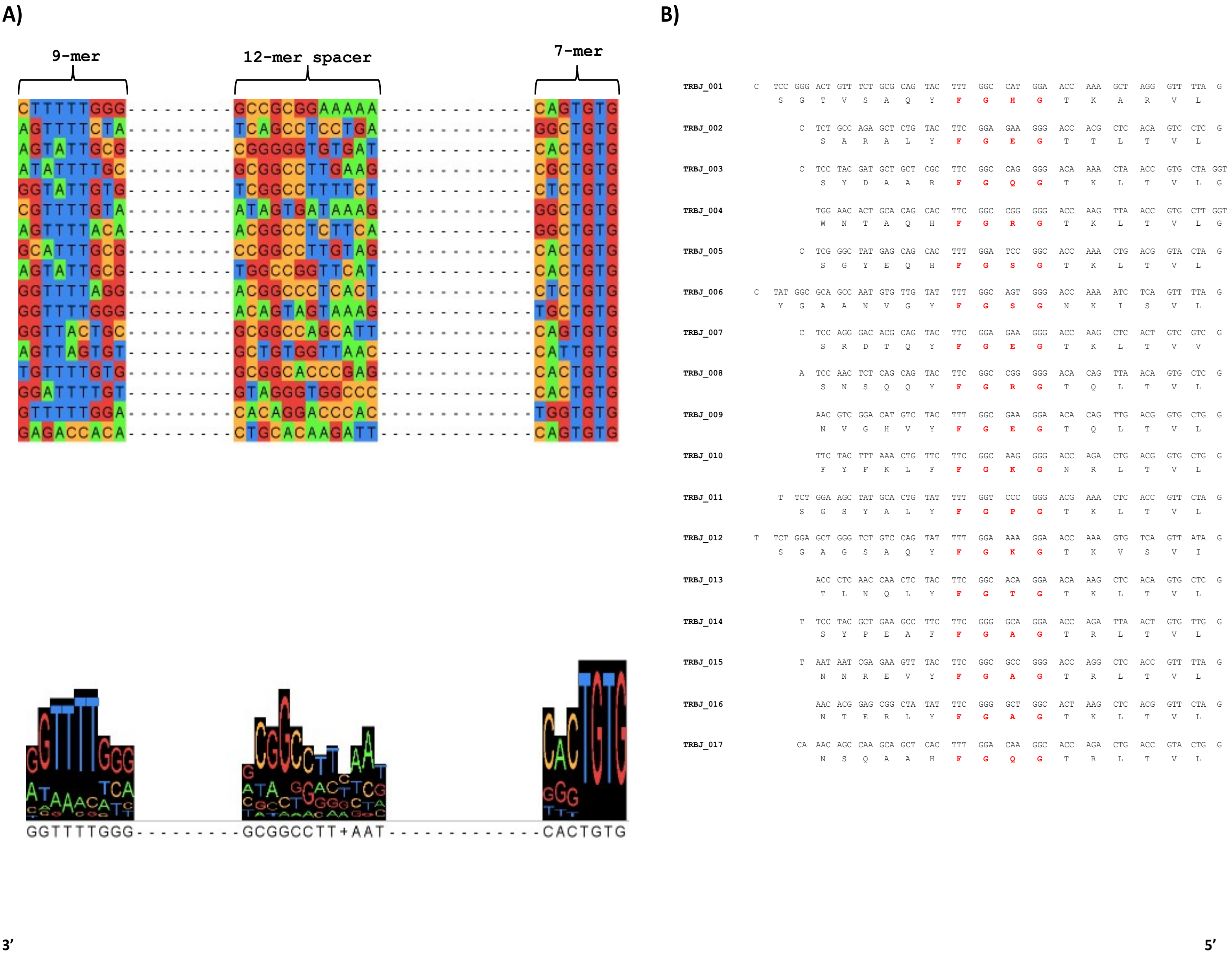

Figure S7. Structural analysis of the *Ambystoma mexicanum* TRBJ genes. A) Alignment of nucleotide sequences of the RSS showing the conserved heptamer and nonamer separated by a 12 bp spacer and considering that the sequences shown correspond to the direct chain 3'-5'. **B)** Alignment nucleotide and amino acid sequences of the 16 identified TRBJ functional genes, the conserved FGXG motif, characteristic of the TRB J-REGION, is red.

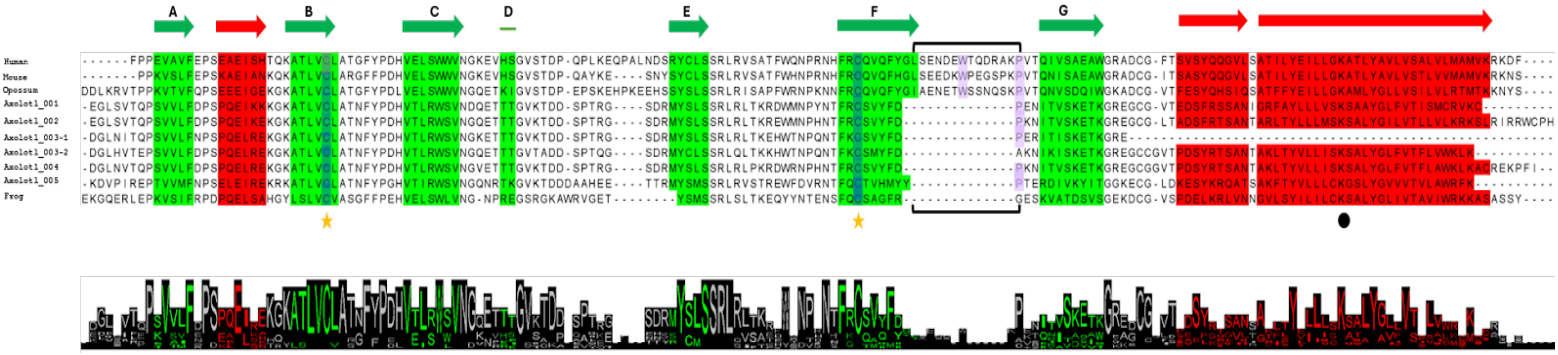

Figure S8. Multiple alignments of TRBC. Sequence comparison of the TRBC FG loop regions among various species (human, mouse, opossum, axolotl, and frog). The position of the F- and G-strands is defined based on the Leu219 TCR structure. The bracketed region defines the elongated FG loop in mammalian species, with well-conserved key residues (Leu219, Trp225, and Pro232) highlighted in purple, forming the hydrophobic core. The yellow stars indicate the two cysteines contributing to the interchain disulfide bond. With a black circle, the conserved lysine throughout all species in the Cβ region is indicated. The arrows in green show the β-strands, and the red ones are the α-helix in the secondary conformation.

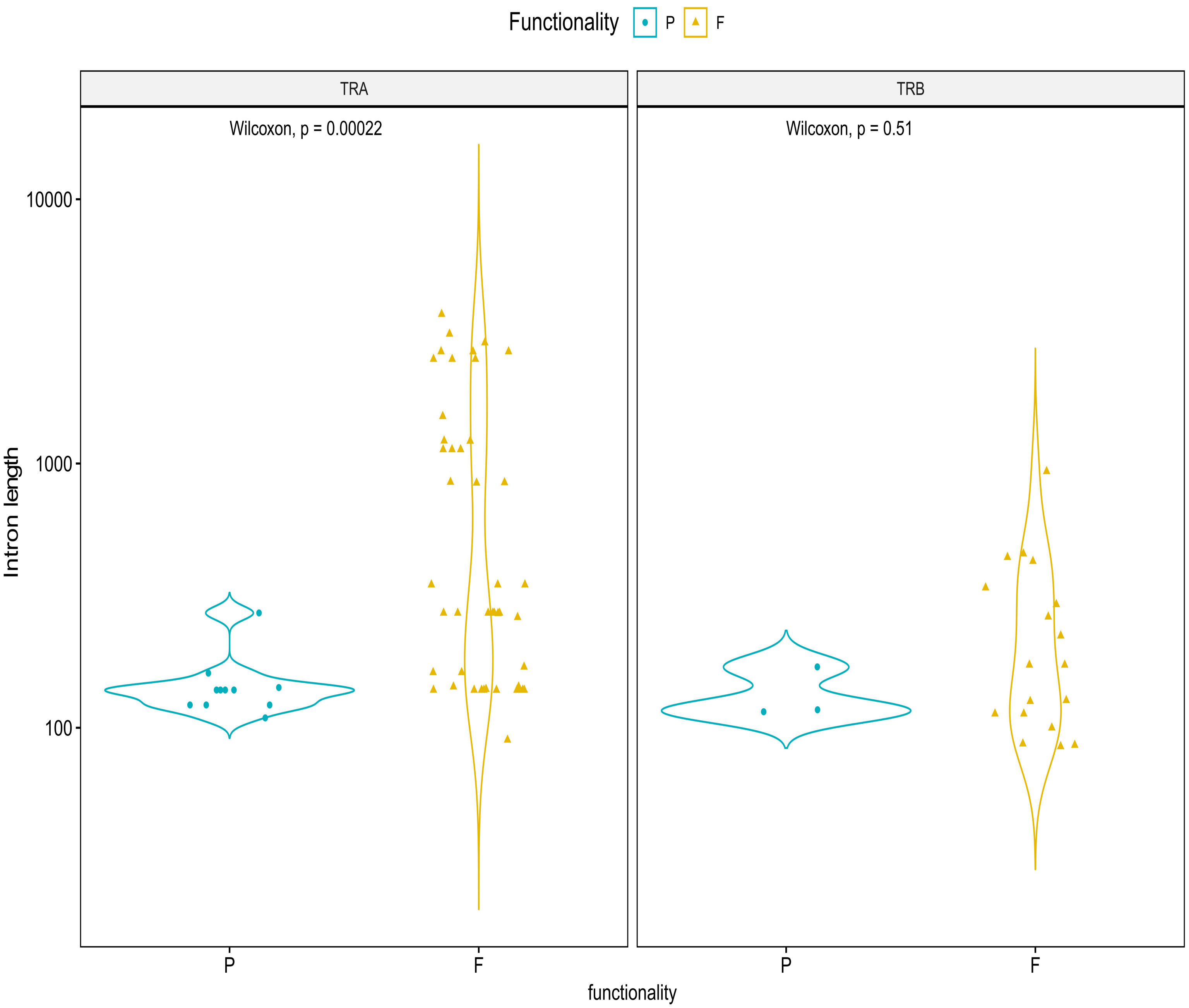

Figure S9. TRA-TRB V-intron. Distribution of intron lengths located between the exons encoding the signal peptide (SP) and the variable (V) region in TRAV and TRBV genes of *Ambystoma mexicanum*. Each point represents an individual gene. In TRAV, functional genes exhibit significantly longer introns compared to non-functional genes (Wilcoxon test, $p < 0.05$). No significant differences in intron length were observed between functional and non-functional genes in TRBV
